# Somatic Symptoms and Sleep Disorders: A Literature Review of Their Relationship, Comorbidities and Treatment

**DOI:** 10.3390/healthcare9091128

**Published:** 2021-08-30

**Authors:** Claudiu Gabriel Ionescu, Ovidiu Popa-Velea, Alexandra Ioana Mihăilescu, Ana Anca Talaşman, Ioana Anca Bădărău

**Affiliations:** 1 Department of Medical Psychology, Faculty of Medicine, University of Medicine and Pharmacy Carol Davila, 050474 Bucharest, Romania; claudiu.ionescu@drd.umfcd.ro (C.G.I.); mihailescu@umfcd.ro (A.I.M.); 2 Department of Psychiatry, Faculty of Medicine, University of Medicine and Pharmacy Carol Davila, 050474 Bucharest, Romania; ana.talasman@umfcd.ro; 3 Department of Physiology, Faculty of Medicine, University of Medicine and Pharmacy Carol Davila, 050474 Bucharest, Romania; anca.badarau@umfcd.ro

**Keywords:** somatoform, insomnia, sleep, somatization, somatic symptoms

## Abstract

This study aimed to investigate the relationship between somatic symptom disorder (SSD) and sleep disorders, following three research questions: (1) How are these disorders correlated? (2) What are the comorbidities reported in these patients? and (3) What are the most effective pharmacological and non-pharmacological treatments for both conditions? PubMed, Scopus, OVID, Medline, and ProQuest databases were searched for relevant articles published between 1957–2020. Search terms included “somatic symptoms disorder”, “sleep disorders”, “insomnia”, “somatoform”, “somatization”, “therapeutic”, “psychotherapy”, and alternative, formerly used terms for SSD. Forty papers were finally included in the study. Prevalence of insomnia in SSD patients ranged between 20.4–48%, with this being strongly correlated to somatic symptoms and psychosocial disability. The most relevant comorbidities were generalized anxiety disorder, depression, fatigue, negative mood, substance use, orthorexia, alexithymia, anorexia, weight loss, poor eating habits, and acute stress disorder. Patients receiving antidepressant therapy reported significant improvements in insomnia and somatic symptoms. In terms of non-pharmacological interventions, cognitive-behavioral therapy (CBT) showed improvements in sleep outcomes, while the Specialized Treatment for Severe Bodily Distress Syndromes (STreSS) may represent an additional promising option. Future research could include other medical and psychosocial variables to complete the picture of the relationship between sleep disorders and somatic symptoms.

## 1. Introduction

In normal individuals, somatic symptoms and sleep disorders are two common conditions that are often connected to acute stress exposure, often in demanding work-related contexts [1]. If persistent, both symptoms can impact quality of life and may require the use of individual counseling and/or organizational supportive systems dedicated to stress prevention and relief [2,3]. Even using these tools, durable alterations of sleep patterns and somatic functioning are still seen in 14–17% of individuals screened for these concerns [4,5,6,7,8,9,10].

A particular category of somatic patients are those suffering of somatic symptom disorder (SSD). According to DSM-V [11], SSD consists in recurring and multiple physical complaints, such as headaches, dizziness, chest pain, abdominal pain, and limb pain. These symptoms do not need to be medically unexplained and may be the consequence of a medical condition. The diagnosis is based on the degree to which a person’s thoughts, feelings, and behavior about their somatic symptoms are disproportionate or excessive. Specifically, one must experience six months of one distressing or disrupting somatic symptom that causes disproportionate and persistent thoughts, feelings, and behavior or that takes up extra time and energy. The symptoms must be clinically significant (i.e., they require medical intervention and impair areas of functioning) and not intentionally produced or feigned. Patients with SSD may report sleep disorders (i.e., patient’s dissatisfaction regarding the quality, timing, and amount of sleep, with resulting daytime distress and impairment [11]) almost twice as frequently (24–32%) than the general population [12,13]. Part of the abovementioned symptoms are particularly important in patients complaining of burdening symptoms, such as pain, where, for example, insomnia can be found in 40–80% of cases [14,15]. The potential of creating a veritable vicious circle between the somatic and sleep disorders, with important effects on the patients’ quality of life, highlights the need to understand the possible role of sleep disorders in the evolution and exacerbation of symptoms among patients with SSD. This relationship should be also investigated from the perspective of the potential role of additional factors, such as psychiatric comorbidities (depression and anxiety), which may frequently occur in this kind of patient [16,17].

In general, the study of sleep disorders in SSD patients is a challenging objective for at least several reasons:

- Definitions and classifications regarding SSD have been a subject of controversy over the past few years. For example, the new DSM-V definition of SSD [11], although being focused on abnormal and excessive thoughts, feelings, and behaviors associated with the burden of somatic symptoms, puts a lower emphasis on their medical explicability and sourcing [18,19,20];
- Despite somatic patients often displaying sleep disorders, not all of them fully meet the DSM-V criteria for SSD. This is partially explained by the DSM-V requiring the symptoms to be persistent (more than six months) and to generate a significant disruption of daily life [11];
- A further cause of bias is represented by the discrepancy between subjective and objective overall sleep quality measures [21]. Although subjective sleep measures have certain advantages (they are less expensive, able to be utilized in large population-based or community-based studies, and very easily standardized), they may over-report sleep duration length among elderly adults or adults in poor health. In contrast, objective measures, despite being more suitable for an adequate evaluation of sleep patterns, are generally costly, invasive, and less tolerated by patients. As a result, 25–50% of patients seek medical attention for sleep disorders [22], while about 20–30% of them develop persisting symptoms, and 71% display a significantly lower quality of life and/or extreme social burden [23,24]; and
- Among SSD patients, a high number of them exhibit conditions, such as depression, anxiety, and fatigue, which themselves are correlated with sleep disorders [25].

As a result of the abovementioned issues, current literature data on the comorbidity of SSD and sleep disorders is still limited. Sleep disorders are not being standardly addressed in the workup of SSD, while, at the same time, they can have consequences for the functionality of the patient and create a vulnerability to further somatic symptoms. In order to clarify the relationship between these two entities, the current literature review aims to answer three questions: (1) How are somatic symptoms in SSD correlated with sleep disorders? (2) What are the most common comorbidities seen in patients with SSD and sleep disorders? and (3) What are the potentially effective pharmacological and non-pharmacological treatment options for both SSD and sleep disorders?

## 2. Materials and Methods

OVID, Medline, PubMed, ProQuest Journals, Web of Science, and Scopus were searched for English language studies on humans published between 1957–2020, assessing somatic symptoms and sleep quality on participants over 18 years old. Papers taken into consideration were peer-reviewed articles, systematic reviews, and meta-analyses corresponding to a level of evidence greater than five in the hierarchy set by the Centre for Evidence-Based Medicine [26] and including the terms “somatic symptoms disorder”, “sleep disorders”, “insomnia”, “somatoform”, “somatization”, “therapeutic”, and “psychotherapy” alongside alternative, formerly used terms for SSD (“somatization disorder”, “medically unexplained symptoms”, “bodily distress syndrome”, “multisomatoform disorder”, and “non-organic pain”).

Exclusion criteria comprised articles centered on (1) obstructive sleep apnea and other similar sleep conditions with possible organic causes, (2) somatic symptoms and complaints caused by chronic organic disorders, (3) pain perception after purposeful sleep restriction, (4) fibromyalgia and related disorders, (5) psychotic disorders and schizophrenia-like disorders, and (6) dementia and related disorders. In addition, when analyzing the third study objective, we limited the search to randomized controlled trials, as we aimed for higher methodological accuracy. The reference lists of retrieved articles were also manually searched for additional information relevant for the study aims.

**Figure 1:**
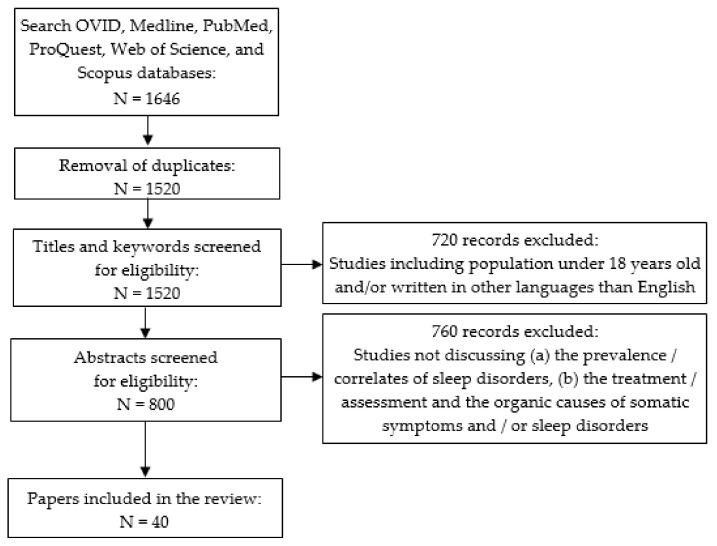
Flow diagram of included articles.

The search was performed according to the PRISMA extension for scoping reviews and the PRISMA-ScR checklist [27]. The first search yielded 1646 hits, which resulted in 800 articles after removing duplicates. Their titles and abstracts were screened for adequacy, defined as addressing both somatic symptoms/somatoform/somatization symptoms together with insomnia/sleep quality/insufficient sleep disorders. Forty relevant articles were finally included in the review (Figure 1). All team members read the full-texts of all the selected articles and agreed upon their inclusion in the review. No additional articles were added after reviewing the references of the selected papers.

## 3. Results

### 3.1. How Are Somatic Symptoms in SSD Correlated to Sleep Disorders?

The search criteria addressing the prevalence, relationship, and prognosis of somatic symptoms in SSD and sleep disorders yielded 15 articles, with sample sizes ranging from 13 to 28,714 (Table 1). Four studies recruited their participants from a psychiatric unit, six from epidemiological studies, and two comprised students, while the other three included healthy volunteers. Eight studies were cross-sectional, while seven were cohort studies.

All studies included a majority of female participants, ranging from 51% to 65%. Most studies included a variety of symptoms, either somatic (somatoform pain, bodily distress, bodily pain, other somatic symptoms) or sleep-related (insomnia, sleep quality, difficulty maintaining sleep, early morning awakening, short sleep duration, and insufficient sleep).

In six studies, insomnia was highly or moderately correlated with the presence of somatic symptoms [28,29,30,31,32,33]. When a larger number of somatic and psychological symptoms were taken into consideration, the prevalence of symptoms in participants with insomnia was even higher [34]. The new onset of insomnia at the one-year follow-up was associated with a higher level of bodily pain and somatic symptoms, among other psychological variables [35].

The severity of insomnia was correlated with the severity of somatic symptoms [32].

This connection extended beyond severity to the presence of somatic symptoms itself and was confirmed during a follow-up study over a five-year time span, which identified insomnia as a predictor of several specific somatic symptoms and physical disorders [36]. However, this effect is not limited to insomnia, as changes in sleep-wake cycle can themselves be associated to the occurrence of somatic symptoms [37].

Despite this seemingly unilateral cause–effect relationship, other authors suggest the existence of a bidirectional connection between insomnia and somatic complaints [30]. More specifically, insomnia could represent a factor in the persistence and aggravation of already-present somatic symptoms as well as in enhancing the psychosocial disability derived from somatic symptoms.

In terms of diagnosis, several studies pointed out that insomnia was more commonly identified than SSD, which is often seen as a secondary diagnosis alongside Axis I psychiatric disorders. From this perspective, a different clinical approach of these conditions and their relationship appears as a critical point to be addressed in future care strategies [38,39,40].

A genetic predisposition with concomitant heritability of somatic symptoms and sleep disorders has been demonstrated, although the contribution of genetic and environmental factors seems comparable (pG = 0.41 − 0.96 vs. pG = 0.27 − 0.40) [32].

Regarding general functioning, insomnia associated with somatic symptoms was correlated to low overall subjective well-being, poorer academic performance, a higher number of doctor visits, and psychosocial disability in four studies [32,34,38,41] and with the lack of motivation, according to one study performed on academic trainees (*r* = −0.18, *p* < 0.05) [33].

An important approach of the relationship between somatic symptoms and insomnia was highlighted in a study where sleep deprivation was performed and in which authors found a direct connection between musculoskeletal symptoms/tenderness and non-REM sleep deprivation [42]. This study must be mentioned as historically important, as it opened the pathway for developing hybrid treatments in SSD, targeting the shared mechanisms of insomnia and chronic pain.

### 3.2. What Are the Most Common Psychiatric Comorbidities Seen in Patients with SSD and Insomnia?

Table 2 outlines 13 studies addressing common psychiatric and psychological comorbidities seen in patients with somatic symptoms and insomnia. The sample sizes ranged from 52 to 148,938. 

The most relevant comorbidities were generalized anxiety disorder, depression [43], fatigue, negative mood [44,45,46,47], substance use disorders (with reports of somatic symptoms and insomnia overlapping withdrawal symptoms) [48], orthorexia nervosa [49], alexithymia [50], anorexia, weight loss, poor eating habits, and acute stress disorder [45,51,52]. An interesting finding was reported by Byers, who established a positive role of cognitive pre-sleep arousal in insomnia patients comorbid with chronic pain as a predictor for insomnia severity (*t* = 2.77, *p* < 0.01). In the same study, pain-catastrophizing did not play a role in insomnia; however, it was independently associated to depression [53].

The reported somatic symptoms varied across studies, from simple somatic complaints to frank SSD and from pain somatoform symptoms to undifferentiated somatoform disorder. Five studies [44,47,52,54,55] were population-based, while the other recruited patients were admitted in hospitals. The identified studies were mainly organized around major psychiatric comorbidities, such as depression and/or anxiety disorders.

### 3.3. What Are the Potentially Effective Pharmacological and Non-Pharmacological Treatment Options for Both Somatic Symptoms and Insomnia?

The selected treatment options for SSD and sleep disorders are reviewed in Table 3 and Table 4. Post-treatment follow-up was on average 12 weeks for pharmacological trials and 9 months for non-pharmacological trials.

Table 3 displays pharmacological treatment options and includes four randomized control trials [56,57,58,59] and a systematic review [60]. The most common pharmacological treatment studied was antidepressant therapy, with various combinations of antipsychotics or other new-generation antidepressants. Participants receiving antidepressant therapy reported statistically significant improvement in insomnia and somatic symptoms [56,57,58]. Other authors suggested that a combination of antidepressant and antipsychotic therapy is better than antidepressant alone in the treatment of SSD and insomnia [59]; however, these combinations should consider the balance between efficacy and side effects.

In current practice, many patients are treated with “off-label” medications that are intended for the treatment of other major comorbidities, like anxiety, depression, and other mental health problems. It remains unclear why medications such as antidepressants are also able to reduce the severity of medically unexplained physical symptoms and insomnia.

Literature on non-pharmacological treatments is summarized in Table 4, including three systematic reviews [61,62,63] and five other trials [64,65,66,67,68]. In these studies, the follow-up period ranged from a few months to one year, with an average number of non-pharmacological therapy sessions of 11 [61]. Cognitive-behavioral therapy was among the most studied non-pharmacological approaches (CBT) for the association of somatic symptoms and sleep disorders, which was investigated in 26 studies. While some authors considered the quality of evidence for a positive CBT effect as being low to moderate [62], other studies report significant differences in the decrease of pain or pain-disability in patients with sleep disorders receiving pain and insomnia-oriented CBT [64,66,67]. Based on the benefits of Pain CBT and Insomnia CBT, two studies looked at a hybrid cognitive-based therapy for pain and insomnia (CBT PI) [66,67]. Both of them showed improvements in sleep outcomes but no difference in pain outcomes when compared to Insomnia CBT or Pain CBT alone.

Short-term psychodynamic psychotherapy (STPP) was considered in one study to have an important benefit for the evolution of somatic symptoms, especially via the boost of self-efficacy involved in their perception [61,67].

An important observation [65] was that the majority of psychotherapies do not focus enough on comorbid depression and learned helplessness, which are both frequent in SSD and insomnia.

The Specialized Treatment for Severe Bodily Distress Syndromes (STreSS) was found in one study to lead to a better evolution of somatic symptoms than enhanced usual care [65].

Mindfulness therapy was reported in another study to display comparable effects to enhanced treatment in improving quality of life and decreasing somatic symptoms [68].

## 4. Discussion

The primary goal of this review on the relationship between sleep disorders and SSD was to reduce uncertainty in the field by answering three main questions relevant for the clinical understanding and management of patients displaying SSD and sleep disorders.

In terms of the correlations between somatic symptoms in SSD and sleep disorders, our findings indicate that patients displaying both conditions have more substantial complaints and worse overall outcomes. Their overall prognosis is correlated with the gravity of the somatic symptoms and sleep disorders, taken separately. This can have implications for choosing therapeutic strategies, as each condition could follow different patterns of evolution and prognosis, eventually leading to a negative loop complex without an integrated solution for both syndromes. Consequently, careful and critical follow-up of these patients is needed in order to identify the most appropriate integrated treatment solutions that could have a good benefit/cost ratio.

Partial evidence supports the bidirectional relationship between SSD and sleep disorders. A possible explanation could be based on the sleep disorders affecting repair processes (thereby contributing to the development of chronic pain), while somatic symptoms-related discomfort could influence the functioning of dopamine and endogenous opioid signaling pathways (thereby affecting sleep patterns). These bidirectional associations could be equally mediated by proteins, such as BDNF (brain-derived neurotrophic factors) or proinflammatory cytokines (IL-1β, TNF-α), which are involved in both pain perception and sleep disorders [69,70,71,72]. We should acknowledge, however, that researchers have still a limited understanding of the complex relationship between SSD and sleep. Beside mediation by neurotransmitters (dopamine, serotonin, or endogenous opioids), alternative theories include the proximity of the pain, sleep, and negative affect and expectation neural pathways or the role played by hyper-arousal states and altered central nervous system information processing [73,74].

Concerning the most common comorbidities seen in patients with SSD and sleep disorders, subjects with both these conditions tend to present multiple symptoms that overlap along their evolution. They are more likely to associate anxiety symptoms, anorexia nervosa, orthorexia nervosa, alexithymia, and catastrophic misinterpretations regarding their somatic condition. Among them, pain and anxiety were reported as risk factors for prognosis [55], while comorbid depression was proven to mediate the relationship between the two variables and to be able to influence nociception and overall prognosis [47]. These findings are important for clinical practice, taking into consideration the multitude of additional variables predicting, maintaining, or mediating the comorbidities of SSD and sleep disorders. From this perspective, substantial research is further needed to identify the best option to address these correlations, especially with respect to symptoms such as alexithymia, anxiety, and orthorexia nervosa. Clinically, the overlap of behavioral indicators of anxiety, certain somatic symptoms, and sleep disorders can also represent a difficulty and requires further refinement of current diagnostic guidelines.

Regarding the potentially effective pharmacological and non-pharmacological treatment options for both somatic symptoms and sleep disorders, we can conclude that, despite several certitudes, there is still room for research, especially about the role of specific pharmacological therapies. Current guidelines are mostly oriented towards targeted-symptoms and comorbidities and less on the overall improvement of SSD in correlation with sleep disorders. Antidepressant therapy was shown to improve somatic symptoms and sleep in several trials in patients with SSD and comorbid depression and insomnia [56,59,60]. Still, more research is needed regarding the efficiency of distinct classes of antidepressant medication. In choosing future treatment options, particular attention should be paid to the cognitive-affective effects of psychotropic medication on encoding, attention, and recall processes, as they may bias the assessment of the results by the physicians and by the patients themselves.

In what concerns non-pharmacological interventions, CBT represents the only psychological approach that has been sufficiently examined regarding its efficacy in SSD patients [75,76]. Its main targets could be represented by the negative affect, the helplessness, and the negative expectations, which are common among patients affected by SSD and sleep disorders. CBT aims the replacement of dysfunctional beliefs, the building of a sleep hygiene routine, the use of healthier strategies towards illness, and restructuring of dysfunctional, symptom-related cognitions [61,62,65,66,67]. STPP has a longer duration of treatment effect compared with pharmacological options, which are generally less acceptable and have a time-dependent efficiency, but has brought no statistically significant differences compared to CBT regarding different mental health conditions [63].

As a whole, our results supplement previous literature findings [77,78] and draw attention to patients with SSD who are in ongoing suffering and may repeatedly seek medical care throughout their lives. In this category of patients, sleep disorders and particularly insomnia can trigger important cognitive, affective, and behavioral consequences and lead to a significant decrease in their overall functionality.

In terms of practical implications, our research highlights several lessons for practitioners in the fields where SSD cases are frequently encountered (Psychiatry, Family Medicine, and Internal Medicine). Firstly, they should consider sleep disorders and their approach in tight relationship with the evolution of SSD. This will help them offer better care to their patients based on treatments focused on both insomnia and pain-related symptoms and to better assess their prognosis. This relationship should also be presented to the patient when discussing their condition, thereby encouraging a better understanding of the underlying mechanisms and precipitating factors. The presence of both conditions could imply a high stress-related vulnerability, which could represent a reason for additional psychological investigations and counseling. Clinicians and patients could thus consider the inclusion of medication in the treatment plan (such as antidepressants) but also non-pharmacological options, such as CBT.

### 4.1. Limitations

This study has several limitations. It was run as a systematic review, so some relevant articles may have not been included. The differences in the definition of sleep disorders and SSD were only partially considered. Other sources of potential bias are represented by differences between clinician-rated and self-reported measures, various cultural backgrounds concerning sample study sizes, and cultural differences in experiencing, presenting, and coping with somatic symptoms and sleep disorders.

### 4.2. Future Research

Multiple studies reviewed in this article provide evidence that sleep disorders add to the decrease of functionality in patients with SSD. In this particular category of patients, sleep disorders require both prevention and intervention, with favorable effects on quality of life and functionality of patients. The development of adequate treatment and prevention strategies is most effective when based on a comprehensive understanding of the underlying causes of co-morbidity. The pathogenesis of sleep disorders in SSD is, however, not yet clear, and further research based on clinical and preclinical studies is needed.

Based on the presented data, investigating the combination of non-pharmacological and pharmacological treatments would be an important direction of research, as both appeared to be more effective than treatment with psychotropic medication alone in anxiety, depression, and in somatization disorders. These effects remained strong and significant up to two years after treatment [79].

Another direction could be represented by the inclusion of new technologies as viable interventions for patients displaying both SSD and sleep disorders. This could include methods such as guided internet-based intervention (iSOMA) for somatic symptoms and related distress and internet-based CBT (CBT-I), both having a wide range of positive effects on the conditions approached in this study and on psychological distress [80].

## 5. Conclusions

Our review aimed to raise awareness for both psychiatric and non-psychiatric practitioners about the importance of prompt detection and treatment of sleep disorders in SSD. Current literature data underpin the importance of assessing both sleep disorders and SSD progression in these patients irrespective of other mental comorbidities. The presence of sleep disorders in SSD can lead to greater severity, longer and disability-generating symptoms, as well as a higher number and severity of associated psychiatric comorbidities. Compared with a targeted treatment model that addresses sleep disorders and SSD separately, a combined treatment model appears to be most effective in terms of costs and prognosis. For comorbid chronic pain and insomnia specifically, a series of pharmacological treatments have had notable results, while CBT remains the best non-pharmacological intervention. Despite the effectiveness of these therapies, future research is critical to assess the value of non-pharmacological and pharmacological treatments in other symptoms of SSD coupled with sleep disorders. Furthermore, describing the pathogenesis and identifying relevant predictive medical and psychosocial variables for the dynamics of SSD and associated sleep disorders are needed to ensure optimal intervention.

## Figures and Tables

**Table 1:** Relationship between sleep disorders and somatization.

Study Authors	Country	Study Design	*n*	Study Aim	Outcomes
Vollrath et al. (1989) [28]	Switzerland	Cohort	457	The association of insomnia with functional syndromes	Insomnia was associated with functional somatic complaints
Kim et al. (2001) [29]	Japan	Cohort	303	The correlation between insomnia and somatic and psychological complaints	The prevalence of insomnia increased with the number of somatic complaints
Aigner et al. (2003) [30]	Austria	Cross- sectional	147	Pain intensity in patients with and without sleep disorders	Sleep disorders were correlated with higher pain in somatoform pain disorder patients
El-Anzi (2006) [31]	Kuwait	Cross- sectional	358	Results of scales regarding depression, anxiety, insomnia, and somatic symptoms	Somatic symptoms were correlated to insomnia
Zhang et al. (2012) [32]	Hong Kong	Cohort	256	Gender differences in patients presenting both sleep disorders and somatization	Insomnia and poor sleep quality was closely associated with pain and somatic symptoms
Schlarb et al. (2017) [33]	Germany	Cohort	2443	The association between somatic complaints and sleep disorders	Somatic complaints are positively associated with subjectively poor sleep quality
Asai et al. (2006) [34]	Japan	Cohort	28,714	The relationship between somatization and sleep disorders	The prevalence of sleep disorders increased with the number of somatic complaints
LeBlanc et al. (2009) [35]	Canada	Cohort	464	Factors related to new-onset insomnia	Higher bodily pain was associated with new-onset insomnia
Zhang et al. (2012) [36]	Hong Kong	Cohort	2316	The longitudinal course of insomnia in patients with mental disorders	Baseline insomnia was associated with chronic pain and poor mental health
Nagane et al. (2016) [37]	Japan	Cross- sectional	135	Sleep-wake patterns as predictors for somatic complaints	The sleep-wake pattern may predict somatic complaints
Tan et al. (1984) [38]	USA	Cross- sectional	100	The association between high emotional arousal and insomnia in SSD patients	SSD were much more common as an additional diagnosis in patients with primary insomnia
Ohaeri and Adeyemi (1990) [39]	Nigeria	Cross- sectional	74	Patterns of somatization symptoms	Insomnia was the most common symptom in patients with somatization
Schneider- Helmert et al. (2001) [40]	The Netherlands	Cross- sectional	51	The association between insomnia and chronic non-organic pain	Insomnia is more than a minor component of chronic non-organic pain
Guo et al. (2017) [41]	China	Cross- sectional	7602	The relationship between depression, pain, and sleep quality	Perceived pain and poor sleep quality were correlated with the number of doctor visits
Moldofsky et al. (1976) [42]	Canada	Cross- sectional	13	The relationship between sleep disorders and musculoskeletal symptoms	The emergence of somatic symptoms is induced by a disorder of non-REM sleep

**Table 2:** Comorbidities in patients with SSD and sleep disorders.

Study Authors	Country	Study Design	*n*	Study Aim	Outcomes
Hartz et al. (2013) [43]	USA	Cross- sectional	148,938	The association between somatic symptoms and sleep disorders	Sleep disorders were positively correlated with somatic symptoms in depressive patients
Gillespie et al. (1999) [44]	Australia	Cohort	3468	Depression and anxiety symptoms in patients with somatization	Somatic symptoms were not genetically or biologically associated to anxiety/depression
Stapleton and Brunetti (2013) [45]	Australia	Cross- sectional	167	The association between depression, somatization, and anxiety and their effect on eating habits	Higher somatization scores were positively associated with depression, anxiety, and poor eating habits
Annagür et al. (2014) [46]	Turkey	Case- control	187	Self-esteem, depressive mood, and their impact on somatic symptoms	Depression and anxiety were linked to chronic pain and sleep disorders
Bekhuis et al. (2016) [47]	The Netherlands	Cohort	2704	The association of somatic symptoms and anxiety/depression	Depression and anxiety were associated with somatic complaints and insomnia
Mol et al. (2005) [48]	The Netherlands	Cross- sectional	193	The pattern of association between depression, somatization, and benzodiazepines craving	Self-reported negative mood and somatization were positively associated with craving
Barthels et al. (2021) [49]	Germany	Case- control	61	The association of orthorexia and depression in SSD patients	Orthorexia levels were elevated in patients with SSD
Lankes et al. (2020) [50]	Germany	Cross- sectional	160	The effect of alexithymia on SSD patients	Alexithymia mediated by negative affect was found in patients with somatoform pain
Davidson et al. (1985) [51]	USA	Cross- sectional	52	The pattern of neurovegetative symptoms in patients with depression	Major depression was linked with insomnia, anorexia, and weight loss in chronic pain patients
Yu et al. (2019) [52]	Hong Kong	Cross- sectional	998	The association of stress, depressive symptoms, and somatization	Stress was associated with depressive symptoms and somatic complaints
Byers et al. (2016) [53]	USA	Cross- sectional	52	The impact of cognitive pre-sleep arousal, catastrophizing on chronic pain, and insomnia	Cognitive pre-sleep arousal predicted insomnia severity in chronic pain patients
Yu et al. (2011) [54]	Hong Kong	Cohort	1433	The pattern of somatic presentation of depression	People with depression were more likely to have multiple medically unexplained symptoms, insomnia, and fatigue
Woud et al. (2016) [55]	Germany/ The Netherlands	Cohort	1538	The impact of catastrophic misinterpretations on SSD	Catastrophic misinterpretations were predictive for somatoform-related problems and new onset SSD

**Table 3:** Pharmacological treatments addressing both sleep disorders and somatization.

Study Authors	Country	*n*	Study Aim	Treatment(s) Duration	Outcomes
Lewis-Hall et al. (1997) [56]	USA	854	The outcome of MDD and somatization with Fluoxetine and TCAs	Fluoxetine vs TCAs, 5 weeks	Significant reductions in somatization and insomnia for both
Saletu-Zyhlarz et al. (2000) [57]	Austria	30	The effects of Zolpidem on insomnia and other psychiatric disorders	Zolpidem vs placebo, 1 week	Improvement in sleep efficiency and somatic complaints
Saletu et al. (2005) [58]	Austria/ Bulgaria	11	The effects of Trazodone on sleep disturbances	Trazodone, 1 week	Increased slow-wave sleep and reduced arousal index
Han et al. (2008) [59]	South Korea	95	The efficacy of Mirtazapine/Venlafaxine in SSD	Mirtazapine vs. Venlafaxine, 12 weeks	Somatization scores decreased from baseline to endpoint for both therapies, results in favor of Mirtazapine
Kleinstäuber et al. (2014) [60]	Germany	2159	The effects of pharmacological therapies on SSD	SSRI vs. SSRI and AP; variable, according to included studies	Low-quality evidence in favor of combined treatment for reducing the severity of somatic complaints

TCA, tricyclic antidepressants; NGA, new-generation antidepressants; SSRI, serotonin selective reuptake inhibitors; AP, antipsychotics.

**Table 4:** The effect of non-pharmacological treatment on somatic symptoms and sleep disorders.

Study Authors	Country	*n*	Study Aim	Compared Interventions	Outcomes
Kleinstäuber et al. (2011) [61]	Germany	1781	The accuracy of STPP for somatization and depression	STPP vs. control	STPP significantly reduced somatic symptoms and depression
Van Dessel et al. (2014) [62]	The Netherlands	2658	The effectiveness of CBT on somatization patients	CBT vs. usual/enhanced care	CBT reduced somatic symptoms at 1-year follow-up but was not more effective compared with enhanced care
Abbas et al. (2020) [63]	United Kingdom	2004	STPP on patients with somatization	STPP vs. minimal treatment	STPP significantly outperformed minimal treatment
Jungquist et al. (2010) [64]	USA	28	The efficiency of CBT for insomnia and chronic pain	CBT vs. control	CBT patients exhibited decreases in sleep latency and increases in efficiency of sleep but no difference in pain severity
Schröder et al. (2012) [65]	Denmark	66	The efficiency of STreSS on patients with somatization	STreSS vs. enhanced care	STreSS group had a greater improvement of the primary outcome than enhanced care
Tang et al. (2012) [66]	United Kingdom	20	The hybrid CBT intervention on sleep and pain outcomes	CBT PI vs. symptom monitoring	Hybrid intervention was associated with greater improvement in sleep, although pain intensity did not change
Pigeon et al. (2012) [67]	USA	21	The efficiency of CBT PI on patients with co-occurring pain and insomnia	CBT PI vs. waiting list	CBT PI produced significant improvement in sleep and disability from pain
Fjorback et al. (2013) [68]	Denmark	119	The efficiency of mindfulness on somatization	Mindfulness therapy vs. enhanced care	Mindfulness therapy was comparable with enhanced care in improvingsomatic symptoms and insomnia

CBT, cognitive-behavioral therapy; STreSS, specialized treatment for severe bodily distress syndromes; STPP, short-term psychodynamic psychotherapy.
